# Quantification of the Early Small-Scale Fishery in the North-Eastern Baltic Sea in the Late 17th Century

**DOI:** 10.1371/journal.pone.0068513

**Published:** 2013-07-05

**Authors:** Aare Verliin, Henn Ojaveer, Katre Kaju, Erki Tammiksaar

**Affiliations:** 1 Estonian Marine Institute, University of Tartu, Tartu, Estonia; 2 Estonian Marine Institute, University of Tartu, Pärnu, Estonia; 3 Estonian National Archives, Tartu, Estonia; 4 Department of Geography, University of Tartu, Tartu, Estonia; 5 Centre for Science Studies, Estonian University of Life Sciences, Tartu, Estonia; Technical University of Denmark, Denmark

## Abstract

Historical perspectives on fisheries and related human behaviour provide valuable information on fishery resources and their exploitation, helping to more appropriately set management targets and determine relevant reference levels. In this study we analyse historical fisheries and fish trade at the north-eastern Baltic Sea coast in the late 17th century. Local consumption and export together amounted to the annual removal of about 200 tonnes of fish from the nearby sea and freshwater bodies. The fishery was very diverse and exploited altogether one cyclostome and 17 fish species with over 90% of the catch being consumed locally. The exported fish consisted almost entirely of high-valued species with Stockholm (Sweden) being the most important export destination. Due to rich political history and natural features of the region, we suggest that the documented evidence of this small-scale fishery should be considered as the first quantitative summary of exploitation of aquatic living resources in the region and can provide a background for future analyses.

## Introduction

Historical perspectives on fisheries and related human behaviour provide valuable information on living aquatic resources and their exploitation/consumption levels, and help therefore to identify historical baselines and set quantitative targets for ecosystem-based management [1]–[4]. Thus, it is essential to broaden the time frame through which we look at fish populations and ecosystems dynamics [5]. Amongst others, this would help to identify patterns and types of fish populations and ecosystems response to various natural and anthropogenic forcings (e.g., [6], [7]).

The importance of fish in the diet of the European human population began to rise in the early Middle Ages (6th to 10th centuries) [8]–[10]. With the gradual depletion of exploited anadromous and freshwater fish stocks in inland water bodies due to increasing number of urban consumers, local fisheries were forced to shift to use the marine fish resources in the coastal seas [8], [11], [12]. For instance, rapid intensification of fishing in the North Sea took place around the turn of the 9th and 10th centuries [11], [13], [14]. During the following centuries, marine fisheries continued to expand their range across the seas of the northern Europe and reaching the coast of North America since the 15th-16th centuries [9], [12], [15]. Along with near extirpations of western European populations of the most valuable anadromous fishes like Atlantic salmon (*Salmo salar*) and European sturgeon (*Acipenser sturio*) already by the first centuries of the second millennia [8], [16], [17], fishing pressure was gradually expanded to the more distant areas like Ireland, Scotland, and also the Baltic Sea to obtain several high-prized coastal and/or migratory species [9], [18].

In contrast to several other areas in Europe and beyond, the quantitative knowledge on fisheries in the Baltic Sea prior essentially to the 19th century is still relatively fragmental. For earlier times, species-based data were recently obtained for the eastern Baltic cod (*Gadus morhua*) population in the central Baltic Sea [19], [20] and for several other fish species in several sub-regions in the north-eastern Baltic Sea and discharging rivers [21]–[23]. The earliest evidence on the northern Baltic Sea fisheries dates back to even the Stone Age where, for instance, remains of nearly 7,000 years old pine bark net floats and stone sinkers were excavated from a Neolithic settlement near the Narva River in the north-eastern Baltic Sea [24]–[26]. Also, bone fragments of several anadromous and freshwater fish species were recorded from broadly the same time period (Mesolithic; [27], and references therein). Danish audit book (Liber Census Daniæ) from the 13th century mentions Narva (Narvia) settlement to be inhabited by fishers and farmers [22]. For the late medieval times, historical Russian records give evidence on the number of weirs used in a river fisheries near the town Ivangorod and upstream near Lake Peipsi during the 15–16th centuries [22], [28]. In addition, there are a few evidences available for the Medieval fisheries in the southern Baltic [29], [30].

**Figure 1:**
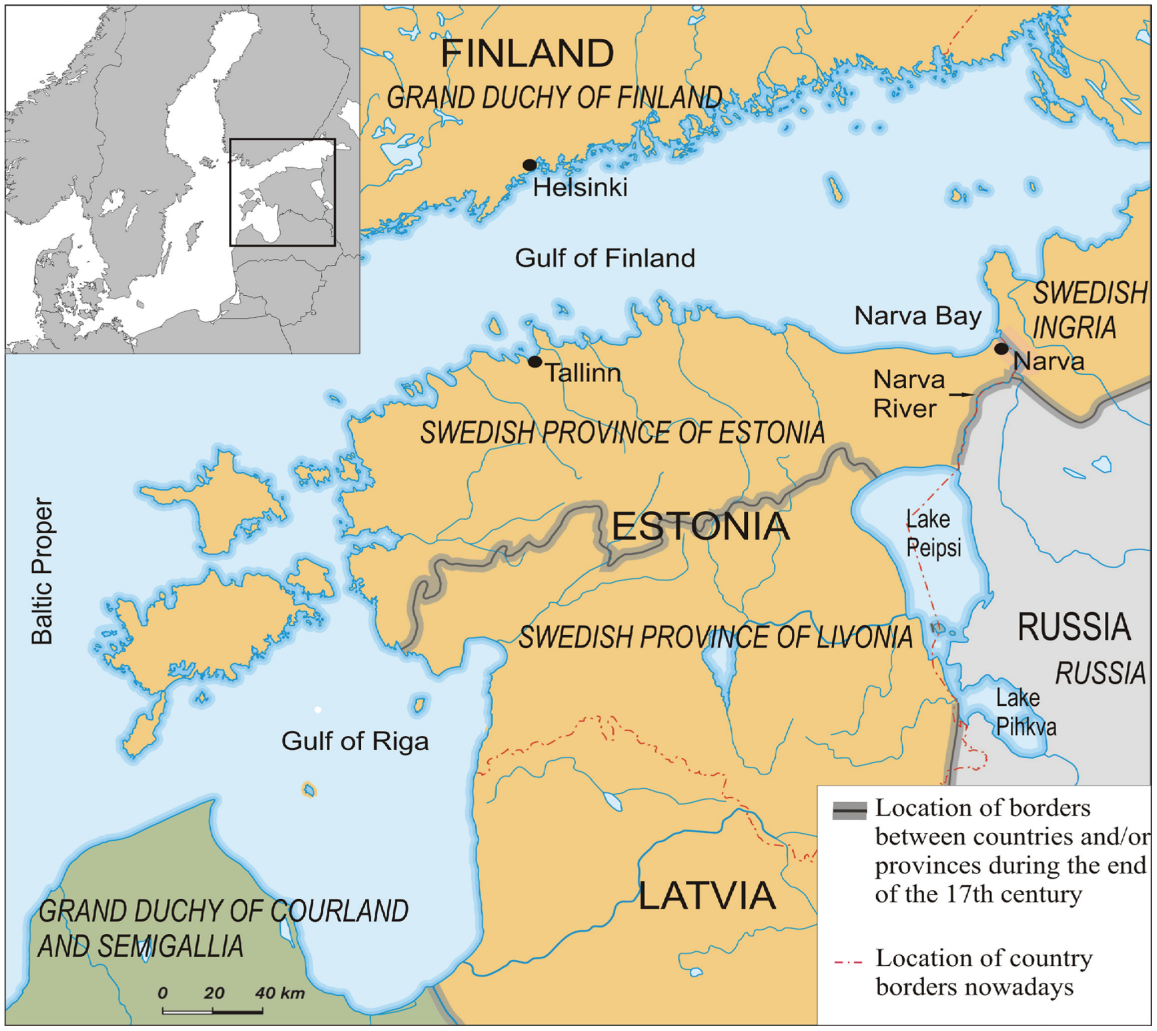
Map showing location of Narva town and surrounding areas. Names of the countries and/or provinces during the end of the 17th century are given in italics.

The study area of the current paper is located in the north-eastern part of the Baltic and extends from the sea (the Narva Bay) all along the western bank of the Narva River and its surroundings to Lake Peipsi with Narva town as a centre (Figure 1). This area is generally very swampy and therefore its human population has historically been sparse [31]. The settlement of Narva was founded in 1223, since the 15th century the importance of Narva as a trade centre significantly rose and its human population started to increase [32], [33]. The Narva River has been historically and is also currently a political border between different powers. During the study period, the western bank and lower reaches of the Narva River including Narva town remained under the Swedish rule. According to estimates, the total number of inhabitants in Narva (without the Swedish garrison) amounted to about 3,000 in the late 17th century [34], [35].

The aim of the current study is to provide a quantitative estimate of local fish catches from the surroundings of Narva during the second half of the 17th century, together with analysis of the monthly composition of the catch. Information on the amounts of fish export is provided, together with species composition and export destinations. The information given should be considered as an early quantitative account on the amounts of fish (by species) marketed in and exported from Narva. As no tax regulation concerning fish trade existed at that time and most of the fish caught were used either by fishers themselves or exchanged for agricultural goods, information on fisheries is very scanty. To compare the results and to obtain a wider perspective on development of local fisheries, available catch data for Narva area from the two more recent periods (the 1930s and 1995–2011) are also provided. Our work is not supposed to give definite baselines for the beginning of the fish resources exploitation, but rather to serve as a documentation of the earliest possible quantitative historical snapshot of the fisheries in an evolving human settlement, which can be in perspective useful in the landscape of setting baselines for this region.

## Materials and Methods

The main source data originate from the customs books from 1662–1703, kept in the Estonian Historical Archives in Tartu (Estonia) as part of the Narva Magistrate’s archive (for details, see Table S1). All together 16 customs books are preserved reflecting the trade in the years 1662, 1666, 1668, 1671, 1672, 1677, 1679, 1689, 1690, 1694–1696, 1698, 1699, 1702 and 1703. In 1704, Russians conquered Narva during the Great Northern War, and some years later (1708) they deported all the citizens into Russia. The available customs books include information about trade and fisheries both in peace and war times, but also during the period of the Great Famine (1695–1697).

Data stored in the Narva customs books provide by no means representative information on the trade and fisheries in the area (the lower reaches of the Narva River, northern Lake Peipsi and the coastal sea being part of Sweden, Figure 1) for the whole period of 1662–1703. For most years, no information could be found on local catches; besides, the records about imported and exported goods reflect only the trade between Narva and Western Europe (incl. Scandinavia) with rare trade records between Narva and Reval (nowadays Tallinn) located at a distance of about 200 km west (Figure 1). It must be also stressed that the Narva customs books contain no information on those goods that passed duty free. Furthermore, there are no traces of any kind of trade between Narva and Russia, although it is well known that several imported goods, among them Atlantic herring (*Clupea harengus*), were delivered further east to Russia.

However, for some years Narva customs records contain very detailed information about local fish catches and fish trade (incl. export and import). So, for the years 1694 and 1695, comprehensive information is provided about fishermen (see below) and amounts of fish traded, together with some important characteristics (e.g. size and fish processing techniques) of different species. Customs books for most other years contain much less detailed information about local catches, and mostly catches are reflected only through exported goods.

Unfortunately, it remains unknown how much fish, fresh or processed, i.e., salted/smoked/dried, did cost on the local market. Therefore, we cannot consider this socio-economic factor in our analysis, but are still able to investigate other important issues such as estimating amounts of local catches (by species) at monthly/annual scale, identifying the source of the fish (freshwater or marine environment), assessing exported amounts (by species) and pointing out major export destinations.

As indicated above, the most complete customs records were available for two consecutive years at the end of the 17th century, 1694 and 1695. These records contain an abundant number of comparable entries (830 and 1033, respectively). Amongst other information, these contain full names of the fishmongers. Due to different scribers and differences in spoken languages, sometimes the name of the same person may vary in the archival material. In such cases, similar name variants were considered to belong to one person. Therefore, a slight underestimation of the number of fishmongers may occur. In addition, less detailed data on local fish catches and fish trade are also available for two earlier years: 1689 and 1690; however, with only 97 and 85 entries, respectively. With the exception of the Baltic herring (*Clupea harengus membras*), information available may contain inaccuracies as in numerous cases archival documents provided no amounts for traded fish species for species like the European whitefish (*Coregonus lavaretus*), ide (*Leuciscus idus*), roach (*Rutilus rutilus*) and ruffe (*Gymnocephalus cernua*). Therefore, the years 1689 and 1690 were omitted from the quantitative analysis.

A variety of different, both volumetric and weight units, used historically to measure fish amounts can be found in the Narva customs books. Significant quantities of traded fish were also counted by number of specimens. These historical measures were converted into common and nowadays used units and expressed in raw wet weight of unprocessed fresh landed fish. Because of lack of any estimated/documented evidence available to the authors, we have assumed that a given volumetric unit contains a similar number of both unprocessed and processed (salted, dried or smoked) fish. The historical measurement units encountered in the archival source material, together with conversion factors and literature sources, are given in Table 1. For the conversion of historical volumetric units into weight units, species average individual weights were applied (Table 2). These individual weight values were taken from the time as far back as possible to resemble broadly similar conditions prior to eutrophication and prior to or at initial stages of commercial fisheries.

**Table 1:** Historical measurement units used in Narva market in the late 17th century.

Unit name	Conversion	Species	Reference
Bunt, bund	10 fish	Northern pike	EAA 1646-2-348 [S1]
L#, lispund	8.29 kg	European perch	[62]
		Freshwater bream	[62]
		Ide	[62]
		Northern pike	[62]
#, pund	0.414 kg	Freshwater bream	[62]
		European perch	[62]
		Northern pike	[62]
S#, skeppund	165.8 kg	European smelt	[62]
		Freshwater bream	[62]
		Northern pike	[62]
Thonne, tonne, tunna	114.8 kg	Atlantic salmon	[62]
		Baltic herring	[62]
		European eel	[62]
		European perch	[62]
		European smelt	[62]
		European whitefish	[62]
		Freshwater bream	[62]
		River lamprey	[62]
		Roach	[62]
		Ruffe	[62]
		Vendace	[62]

**Table 2:** Species average weight applied in the calculation of Narva fish trade in the late 17th century.

Species	Averageweight (g)	Reference
Atlantic salmon	10000	[63]–[66]
Baltic herring	22	[63]
Burbot (rivers)	200	[67]
Burbot (Lake Peipsi)	1000	[67]
Crucian carp	500	[67]
European eel	700	[67]
European perch	200	[63]
European whitefish (Lake Peipsi)	600	[67]
Freshwater bream	700	[63]
Grayling	250	[67]
Ide	400	[63]
Northern pike	700	[63]
River lamprey	60	[67]
Roach	200	[63]
Ruffe	12	[68]
Stone loach	12	[67]
Vimba bream/European whitefish	300	[63]

**Figure 2:**
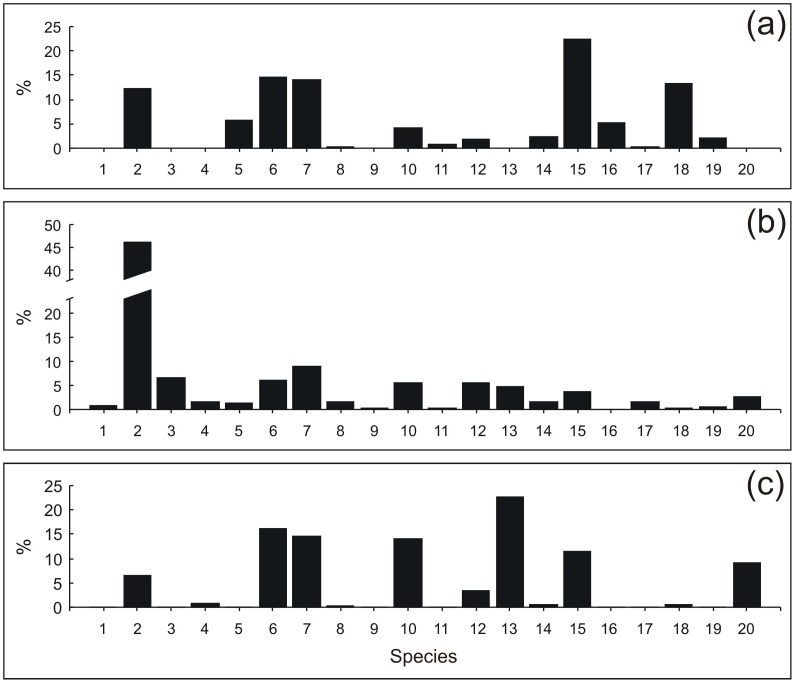
The relative share of different species in fish catches during different time-periods. Narva town fish market, 1694–1695 (5a), the eastern Gulf of Finland and Lake Peipsi fisheries, 1930; 1935–1939 (5b), the eastern Gulf of Finland and Lake Peipsi fisheries, 1995–2011 (5c). Numbers on the category axis represent following species or species groups: 1- Atlantic cod; 2- Baltic herring; 3- European sprat; 4- Burbot; 5- European eel; 6- European perch; 7- European smelt; 9- Flatfishes (European flounder and turbot (*Scopthalmus maximus*); 10- Freshwater bream; 11- Ide; 12- Northern pike; 13- Pike-perch; 14- River lamprey; 15- Roach; 16- Ruffe; 17- Salmonids (Atlantic salmon and sea trout); 18- Vendace; 19- Vimba bream/European whitefish; 20- Other species.

Catch data for years 1930–1939 are extracted from Estonian monthly fisheries magazines and for 1995–2011 from archives of the Estonian Marine Institute and from the national official catch statistics. As Atlantic salmon and sea trout (*Salmo trutta*) were not identified to a species level in the archival records, we have combined both species to the category salmonids in the contemporary datasets (Figure 2). Other fish categories include mainly species like ruffe for Lake Peipsi, and eelpout (*Zoarces viviparus*) and Prussian carp (*Carassius gibelio*) in the modern day Gulf of Finland (Figure 2).

## Results

### Local Exploitation

The retrieved Narva customs books confirm that fish was an important food source for local people in the late 17th century. Probably over 90% of the local catch was sold in the Narva market and therefore is considered to be consumed locally. The total local consumption amounted to ca 180 and 230 tonnes in the years with the most complete accounts, i.e. 1694 and 1695, respectively (Table 3). The combined number of fishermen and fish merchants in 1694 and 1695 can be estimated at 420 and 470, respectively. Altogether one cyclostome and 17 fish species were exploited and consumed. Among these, six species dominated constituting about 86% of the catches. These were the freshwater species European smelt (*Osmerus eperlanus*), European perch (*Perca fluviatilis*), roach, ruffe and vendace (*Coregonus albula*) and one marine species, the Baltic herring. The individual share of each of them exceeded 10% of the total weight of the fish sold in the market. The annual proportion of regularly occurring species in the catch, like freshwater bream (*Abramis brama*), European eel (*Anguilla anguilla*), ide, northern pike (*Esox lucius*) and vimba bream (*Vimba vimba*)/European whitefish (see below) fluctuated between 1% and 10%. Finally, there were several relatively rare species such as burbot (*Lota lota*), crucian carp (*Carassius carassius*), grayling (*Thymallus thymallus*), stone loach (*Barbatula barbatula*) and also European whitefish, which were sold only occasionally. The most valuable species such as salmon and the cyclostome, river lamprey (*Lampetra fluviatilis*), were mostly exported to the capital city of the Swedish kingdom Stockholm and the Hanseatic town Lübeck (see below) and therefore were sold in the local market seldomly.

**Table 3:** Amounts of locally consumed (LC) and exported species (kg) in Narva (1694–1695).

Species	1694			1695		
	Total	LC	Export	Total	LC	Export
Atlantic salmon	1791	543	1248	114	0	114
Baltic herring	27648	27189	459	25922	25807	115
Burbot	9	9	0	25	25	0
Crucian carp	33	33	0	33	33	0
European eel	9614	5481	4133	15413	10319	5094
European perch	19664	19664	0	44639	44639	0
European smelt	7434	7434	0	54013	54013	0
European whitefish	12	12	0	707	707	0
Freshwater bream	5290	5290	0	12855	12855	0
Grayling	59	59	0	21	21	0
Ide	1764	1764	0	1812	1812	0
Northern pike	3066	2609	457	4856	4807	49
River lamprey	6350	0	6350	3680	0	3680
Roach	38597	38597	0	59590	59590	0
Ruffe	22137	22137	0	792	792	0
Stone loach	61	61	0	0	0	0
Vendace	49551	49479	72	8840	8840	0
Vimba bream/European whitefish	4596	4596	0	4965	4965	0
Species total	197676	184957	12719	238277	229225	9052

About 84% of the locally exploited fish was brought to and sold in the local market by people bearing Russian names. Marine fishes like the Baltic herring were traded mainly by people of Swedish origin (14% of the total trade). According to traders’ names in customs records, only a minor part (less than 2%) of the fish trade was conducted by other ethnic groups such as Estonians, Germans, Ingrian Finns and others (Table 4).

**Table 4:** Amounts of locally consumed species (kg) traded by different ethnic groups in Narva (1694–1695).

Species	Russian		Swedish		Other	
	1694	1695	1694	1695	1694	1695
Atlantic salmon	0	0	545	0	0	0
Baltic herring	4400	8712	22674	16337	115	758
Burbot	9	25	0	0	0	0
Crucian carp	33	33	0	0	0	0
European eel	5439	10319	42	0	0	0
European perch	18946	43290	718	918	0	431
European smelt	7204	53037	230	0	0	976
European whitefish	12	707	0	0	0	0
Freshwater bream	5269	12665	21	21	0	169
Grayling	53	21	5	0	0	0
Ide	1352	1658	412	34	0	120
Northern pike	2518	4583	77	77	14	147
Roach	30182	54571	7227	3929	1188	1090
Ruffe	20319	635	1516	121	302	36
Stone loach	61	0	0	0	0	0
Vendace	47872	8840	0	0	1607	0
Vimba bream/European whitefish	2511	3165	2085	1800	0	0
Species total	146180	202261	35552	23237	3226	3727

### Seasonality and Types of Processing of Marketed Fish

The most intensive fishing took place in spring and early summer. However, winter was also an important season with under-ice fisheries, when prolonged periods of sub-zero temperatures allowed transport of frozen fish over long distances.

Roach was the most important species in the market, contributing approximately 25% of the local fish consumption. The fish was mostly sold during its spawning season in spring and early summer. However, roach was also present in the market in a limited amount during the rest of the year. In winter and spring, roach was marketed fresh or even alive, but salted and/or dried during summer and autumn.

**Figure 3:**
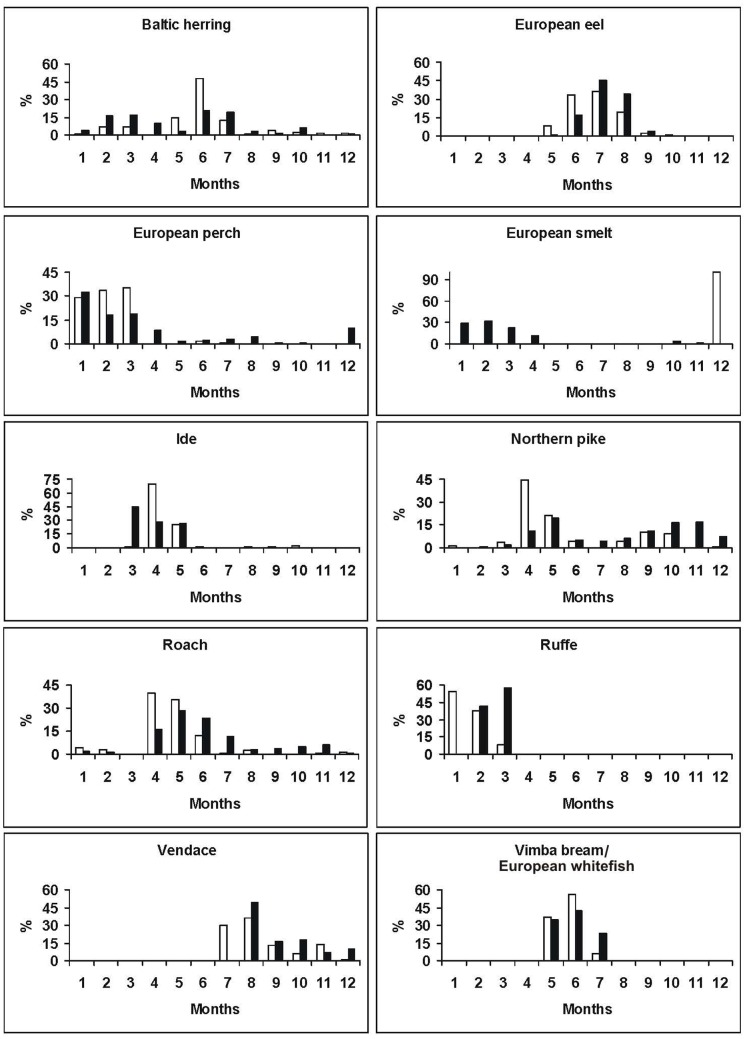
Monthly trade dynamics of different fish species in Narva fish market. Expressed as a percentage of the total traded species raw biomass, for the years 1694 (empty column) and 1695 (black column).

**Figure 4:**
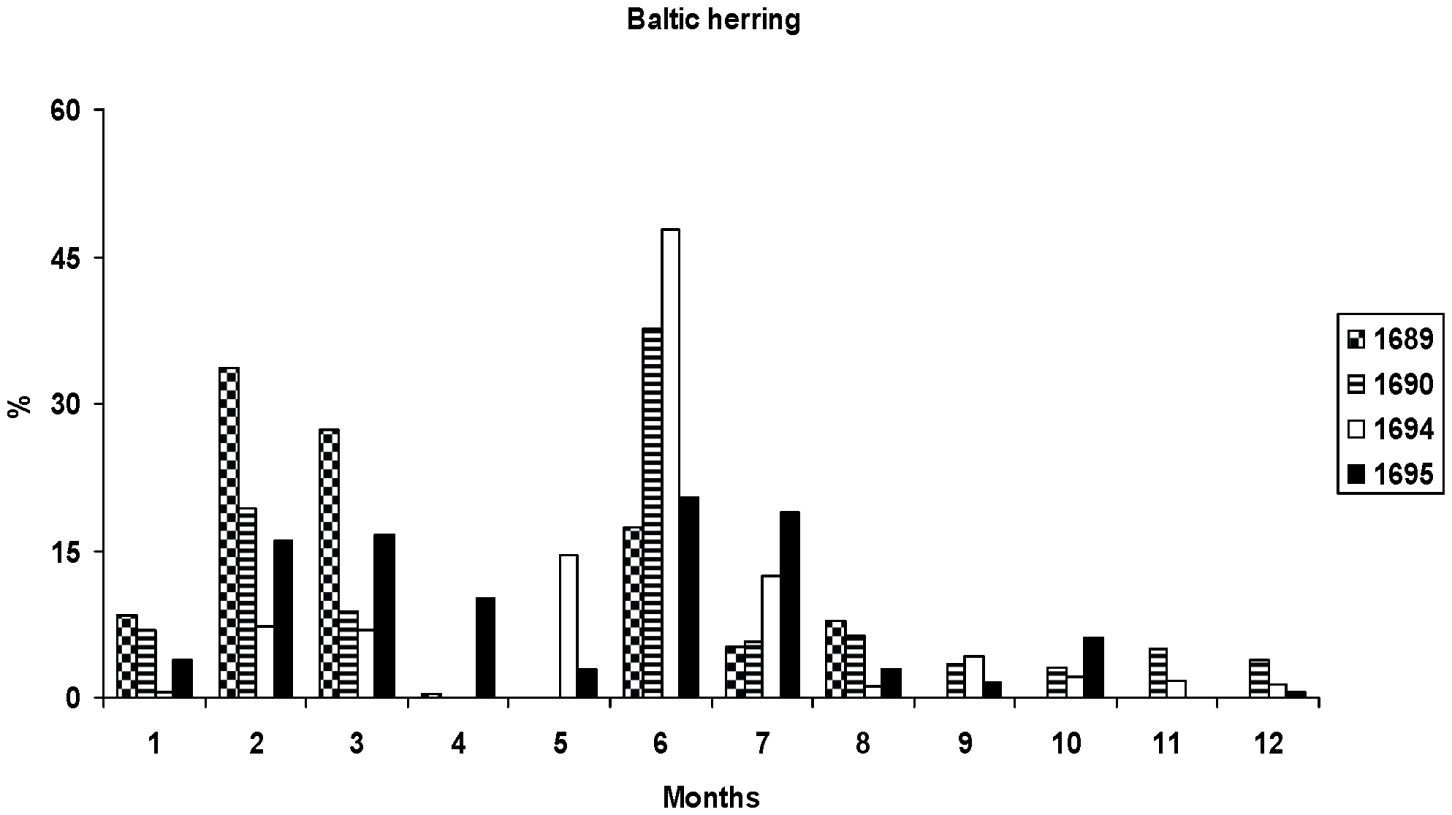
Monthly trade dynamics of the Baltic herring in Narva fish market. Expressed as a percentage of the total traded species raw biomass, for the years 1689, 1690, 1694 and 1695.

The remaining five most important fish species–the Baltic herring, European perch, ruffe, European smelt and vendace–accounted each for about a tenth of the amount of the marketed fish. In case of ruffe, European smelt and vendace, great variations occurred between quantities marketed in 1694 and 1695. The Baltic herring had annually two main marketing seasons: winter (February–March) and spring/summer (May–July) (Figures 3, 4). European perch was mainly sold in the winter season, less during spring and summer. Vendace was mostly sold in late summer, in July and August, but also in considerable amounts during the autumn months. The Baltic herring and European perch were sold fresh during the cold season (from winter to early spring), and marketed as salted and/or dried from late spring to autumn. Ruffe and European smelt were marketed only as fresh during the cold time (from October to April) with a peak in the winter months (Figure 3). Vendace arrived in the market as salted fish packed in barrels.

Representatives of the regular species group appeared in the market mainly seasonally, associated probably with their migrations close to the coast or spawning events. Freshwater bream, ide, northern pike, vimba bream/European whitefish were marketed predominantly in spring and early summer and were brought to the market in relatively small quantities as fresh every day. European eel was sold almost exclusively during summer months and marketed as fresh. During summer freshwater bream was usually marketed as salted or in smaller amounts as dried and packed in barrels. Very small amounts (usually a few dozens) of northern pike were sometimes even sold alive or as dried. Northern pike had also one less important marketing season in autumn, from September to November (Figure 3).

A group of irregularly occurring species was brought to the market occasionally and usually in relatively small amounts. Crucian carp and stone loach were sold at several occasions during the spring season, crucian carp also in summer. Grayling arrived in the market mainly during autumn in September and October, and burbot and European whitefish during late autumn and winter months from November to March. The traded quantities did not exceed some dozens of specimens for burbot, crucian carp and grayling. Stone loach was usually sold in quantities consisting of several hundreds of fish. Salmon reached the local market only in one occasion: in February 1694, almost 5 barrels (545 kg) of salted salmon was sold by three Swedish-speaking persons. All irregularly occurring species except salmon were marketed exclusively as fresh.

Based on the results above and the prevailing political situation during the late 17th century, supplemented by information and knowledge on the likely fishing methods applied at that time, we estimated the approximate fishing area that supported the fish market in Narva. We suggest that this area includes coastal waters of the Gulf of Finland extending a only a few kilometres towards the sea, and inland freshwaters by including northern part of Lake Peipsi and most likely only the western coast of the Narva River.

Because of uncertainties in the fishing area in the 17th century and availability of catch statistics in the 20–21th century, comparison of catch quantity might be not the best way on how to locate the 17th century data into historical perspective. Our calculations indicate that annual fish catches from the eastern Gulf of Finland and Lake Peipsi amounted to 3800 tonnes in the 1930s and to 7600 during 1995–2011. However, we must consider that the modern fishing data originate from much larger area than in the 17th century, however, the exact extent is very difficult to quantify. The percentage of different fish species in catches should give much better and reliable picture. It appears that the Baltic herring, European perch, European smelt and freshwater bream dominate in fisheries across all the different time-periods investigated. Proportion of European eel, roach and vendace was substantially higher in the 17th century while pike-perch (*Sander lucioperca*) played substantial proportion in the turn of the 21st century. The share of the Baltic herring and European sprat (*Sprattus sprattus*) was substantially higher in coastal fisheries in the 1930s compared to that during the other two time-periods. However, the number of species exploited by commercial fishery was substantially lower during the most recent time period compared to the end of the 17th century and the 1930s (Figure 2).

### Fish Export

**Figure 5:**
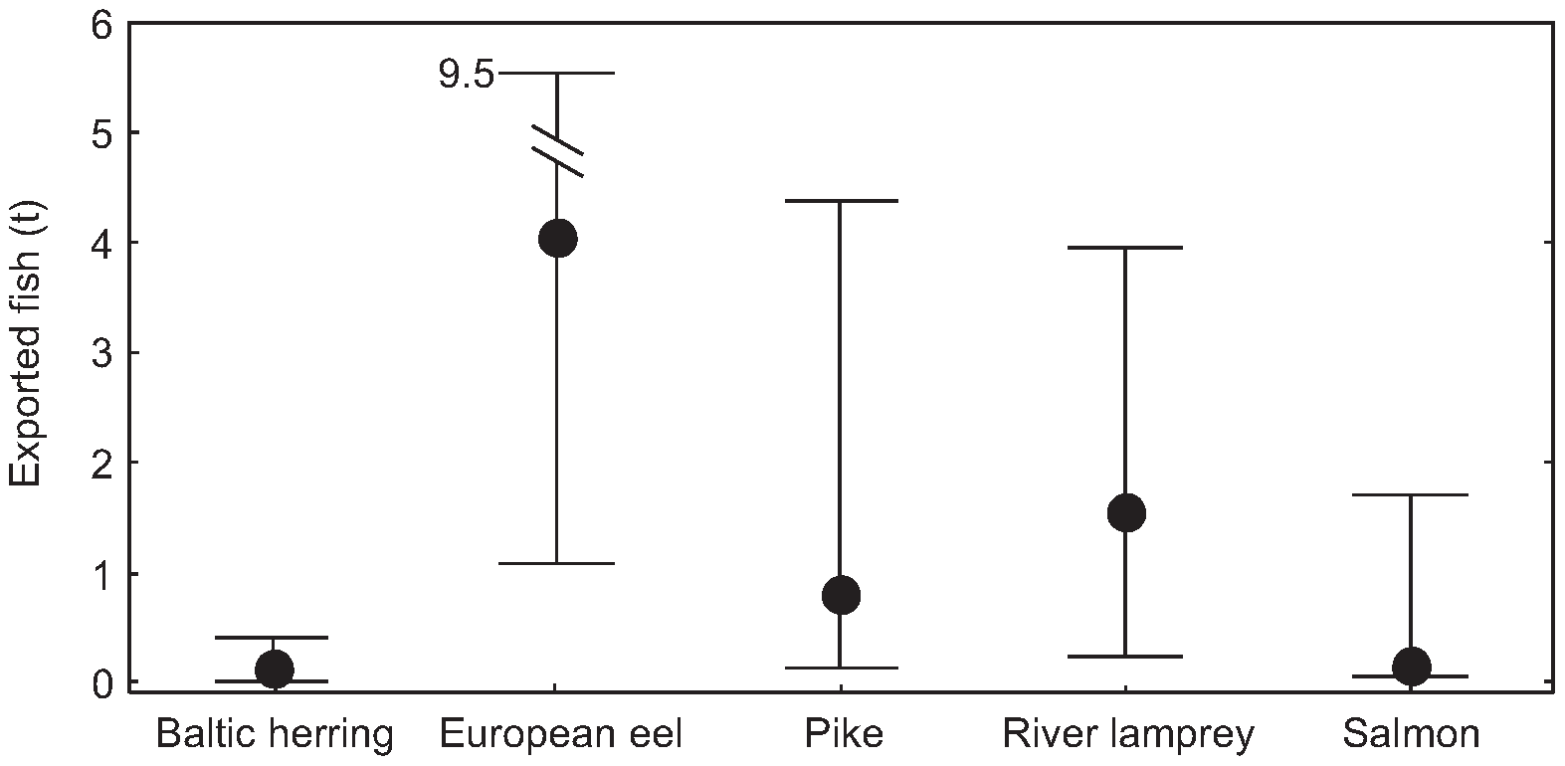
Export amounts of five most important species from Narva during the years 1662–1699. Expressed as median ±50% quartiles.

Archival data related to the fish export from Narva are available for 12 years in the late 17th century (Figure 5). Generally, the export from Narva did not exceed a few dozens of tonnes annually and was about one order of magnitude lower than the local fish consumption. The record level of export amounted to 32 tonnes in 1679.

The most important export destination was Stockholm (Sweden), receiving more than 70% of the total fish export in terms of biomass, followed by Lübeck (Germany) with 27% and Reval (nowadays Tallinn, Estonia) with 1.5%. The other, only very rare export destinations found in the customs books were Amsterdam (The Netherlands), England, Gävle (Sweden), Holstein (Germany), Maholm (nowadays Mahu, Estonia), Riga (Latvia), Stettin (nowadays Szczecin, Poland), Tolssburg (nowadays Toolse, Estonia) and Wyborg (nowadays Vyborg, Russia) with less than 0.5% in terms of exported fish biomass.

In total one cyclostomes and eight fish species were mentioned in the customs books export lists. European eel was far the most important export object, accounting for almost half of the total weight. Other more important export species were northern pike, river lamprey and Atlantic salmon. During the recorded 10 years from the period 1662–1699, almost 60 tonnes of European eels was exported from Narva, the majority (58%) was headed to Stockholm and a smaller part (39%) to Lübeck. Other destination ports received less than 1% each. More than four-fifths of northern pike (81%) was exported to Stockholm, virtually all the remaining part (19%) to Lübeck. The major export destinations for river lamprey were Stockholm (71%), Lübeck (20%) and Reval (7%).The bulk of Atlantic salmon (87%) was exported to Stockholm and practically all the remaining quantity (>12%) went to Lübeck.

The most intensive export associated with European eel, northern pike and Atlantic salmon occurred during the summer months. Two peaks are observable in river lamprey exports: in spring (May–June) and autumn (September–October). No export occurred during the period of ice cover lasting from December to March.

Only processed fish was exported. For instance, European eel, river lamprey and Atlantic salmon were usually shipped salted in barrels, river lamprey and Atlantic salmon (European eel in minor amounts) also in significant quantities as dried fish. Almost all northern pike was exported dried. Some dozens of Atlantic salmon were recorded to have been smoked. Some barrels of river lamprey were described as preserved (*eingemachte*) fish and in one occasion 150 river lampreys as smoked (*röchta*) fish.

The amounts of exported Atlantic salmon fluctuated significantly between the years: from some dozens of kilograms to several tonnes per year (Figure 5). An interesting case of a large-scale Atlantic salmon transit was recorded in June 1690 when a ship with a cargo containing 29 barrels (more than 3.2 tonnes) of salted fish was forced to wait for the weather to improve in the port of Narva. Unfortunately, the vessels’ departure and destination points are not stated in the customs records.

As to the less important export fishes, the archival documents with 10 written records confirm that during the observed period the Baltic herring was exported to Stockholm in relatively small one-time quantities (1–5 barrels or 100–600 kg) as salted or dried fish. Dried freshwater bream was exported to Stockholm in two cases in June 1679 and once to Gävle in June 1689. Vendace, European smelt and European perch were mentioned in export lists only once. In 1694, less than three quarters of a barrel (72 kg) of salted vendace was shipped to Stockholm. In September 1668, one ship’s pound (skeppund; 166 kg) of dried European smelt was exported to Stockholm. In June 1689, 74 pounds (31 kg) of dried European perch was exported to Gävle.

## Discussion

The current study provides archive-based quantitative information on local fish catches by species for an area extending from the sea (NE coast of the Gulf of Finland) to the northern part of the Lake Peipsi, by including most likely the west coast of the Narva River only and also any small freshwater bodies in the region. We have found that most of the fish of this area probably originate from the adjacent freshwater bodies. Essentially because of geopolitical reasons and natural conditions prevailing in this region, Narva and adjacent areas in the south were relatively unimportant until the 15th century. One of the most important events in this regard was the Livonian War, after which the importance of Narva in the trade between Russia and Sweden substantially increased [33], [36]. Thus, our study should be considered as characterising the situation and related human habits in the evolving stages of fisheries, where the exploitation rate of the species (perhaps excluding sturgeon only, see also below) hasn’t reached high and unsustainable levels as yet [37]. During this period, close to 5% of the 10,000 estimated inhabitants in the region (E. Tammiksaar, unpubl. data) were involved in fish catch or trade. It is important also to mention that there is no evidence on earlier archival fisheries records from this specific area: there was no tax regulation and therefore this field was relatively unprofitable with most of the fish caught being consumed by local people [38]. However, there is some evidence on fisheries from the nearby region at approximately similar time-period, which broadly corresponds to that of our results [22], [23].

It is important to mention that archival documents retrieved by us do not provide any information on the source of caught/traded fish. As the Baltic Sea is a brackish water body and freshwater fish can be also very abundant in the coastal sea (e.g., [39]), for interpreting the origin of the fish we used knowledge on the spatial occurrence of different ethnic groups at those times and applied this information to evaluate their access to different water bodies. Archival documents confirm that almost all the Baltic herring was traded by Swedish-speaking people, whose settlements were located near the seacoast (see also Figure 1). A similar situation prevailed for freshwater fishes: these were almost exclusively traded by Russian-speaking people. Russians invaded the northern coast of Lake Peipsi and to some extent also the western coast of the Narva River during the 15th–17th centuries [40] with the main purpose of making their living based on utilising the relatively rich fish resources of this region [31], [33], [38], [41]. They were experienced fishermen, who mainly caught fish from freshwater bodies. However, to assess the exact source of freshwater fish, i.e., whether fish was caught from the Narva River or Lake Peipsi, was a challenging task.

The origin of several fish species in the catch is worth discussion. One of them is vendace. As the peak season of the historical vendace trade corresponds to that of Lake Peipsi vendace fishery nowadays [42], traders of this fish were never involved in marine fish trade and the species was sold exclusively by Russian-speaking persons, we suggest that vendace originates from Lake Peipsi. Based on the same logic we suggest that also European smelt sold in the fish market originated from Lake Peipsi. However, several species were traded by both Russian and Swedish fishmongers and therefore we assume that these fishes probably originated both from Lake Peipsi and the Narva River as well as smaller water bodies near the town. These are freshwater bream, European eel, ide, European perch, northern pike, roach and ruffe. Most of them probably originated from Lake Peipsi. Our assumption is based on the ethnic information on fish traders and co-occurrence of typical Lake Peipsi fish (vendace) in trade. However several cyprinid and percid species were sold fresh by both Swedish and Russian fishmongers, and therefore, these were supposedly caught from streams and rivers in the vicinity of Narva (and not from Lake Peipsi).

Herring has been the most important commercial fish in the Baltic for centuries (e.g., [43]–[45] and we suggest that this was the only species directly caught from the sea in our study. The species was exploited both in summer and during winter. While seine fishing during the ice-free season has been practiced in the northern Baltic Sea for several centuries (perhaps since the 15th century [46]), it has been unclear since when this method has been in use in the under-ice fisheries. Our research suggests that in northern Baltic Sea the under-ice seine fishing for herring has been in use at least since the late 17th century. The ecological basis for the Baltic herring winter fisheries needs to be explained further. It is known that clupeids overwinter in deeper water layers [47] and therefore should not be available for seine fishing under ice in coastal waters during the winter. However, due to the bottom topography of the area, (characterised by sharp slopes near the coast) clupeids may be available for under-ice fisheries also during the winter, but especially towards the spring, when fish already start to perform diurnal vertical migrations, and during sunny days [48], when the diurnal migration is clearly pronounced [47]. Although the under-ice catch likely also contains European sprat (e.g. [48]), in the archival records no indication that sprat was either locally marketed or exported could be found.

Disputes about the content of the archival documents either on obviously missing species or assembling two similar species together into one species category are not new. Such a phenomenon has been observed earlier also concerning the Baltic Sea [44]. In the current study, we have identified several such cases, like potentially combining European whitefish and vimba bream, and Atlantic salmon and sea trout into one entry. While whitefish occurred as a common species in the market entries, the archival documents did not contain any information on the trade of vimba bream. However, vimba bream was mentioned as a very important commercial fish in the neighbouring area of Luga and Koporye bays in the 18th century [22]. Therefore, we propose that because of the similarities between their traders ethnic information, trading season, processing information, and partly also external appearance, together with supporting ecological information available from contemporary times, European whitefish and vimba bream may potentially have been recorded in the archival documents under the same name as *syk/syck*. Accidental inclusion of sea trout into Atlantic salmon catch/trade information was possible, but probably not common practice, as due to high river flow rates, the salmonid spawning areas of the Narva River are almost entirely suitable only for salmon. Therefore, salmonid catches of the Narva River consisted likely predominantly of Atlantic salmon in previous centuries [49]. Judging by the subfossil bone finds, sturgeon species like Atlantic sturgeon (*Acipenser oxyrinchus*) and European sturgeon (*A. sturio*) [50] represented a significant part of the Baltic fisheries up to 8th-10th centuries [27], [51], after which its importance started to decline [52]. As no sturgeon catches were reported in the archival sources investigated in this study, we assume that this very valuable fish might already have been extirpated. Also, the stylized sturgeon pictured on the coat of arms of Narva town in the 14th centuries was replaced with the graylings in the 15th century [22], [33]. This might serve as another evidence of disappearance of sturgeon in this area. And finally, there is another missing species from 17th century records – pike-perch – which is currently an important commercially exploited fish in this area (Figure 2).

The fish export volumes identified within the current study, reaching maximally some dozens of tonnes per year, were relatively insignificant if compared to other European fisheries which exported thousands of tonnes of marine or migratory fish annually [4], [43], [53], [54]. However, the export consisted predominantly of most valuable species, whose demand among the European urban consumers always exceeded supply [8] and which was increasingly met through long distance transport by the 13–14th centuries [14]. While most of the locally caught valuable fish taxa (European eel, river lamprey, northern pike, Atlantic salmon) were all exported, the nowadays non-commercial stone loach was for some reason not included in the export list. However, this species was valued as a good food fish during previous centuries in Germany and Russia [22], [55] and was kept as a delicacy in ponds in Sweden in the 17th century [56].

Judging by archaeological evidence, practically all known gear types of small-scale fisheries such as trap and seine nets, fish weirs, fish spears and hook lines were in use in local fisheries in the Gulf of Finland already before the end of the Stone Age [57]. However, and unfortunately, due to uncertainties involved in fishing areas, exact number of fishermen, trade peculiarities and different fishing gear distributions, values on the catch per unit effort (CPUE) can not be calculated within the current study. This also makes comparisons with the more recent situation more complicated and related interpretations weaker.

The current end-17th century fishery in the north-eastern Baltic Sea should be interpreted as an example of evolving peripheral settlement bearing several features of medieval fisheries. Because the area was sparsely populated, intensity of the local fishery had not yet reached unsustainable level and therefore exploited mainly freshwater fish populations, which were a more easily accessible resource. This is similar to that in Western Europe during the early Middle Ages. By that time, there are several evidences on local extinctions of exploited living resources from various regions globally (e.g., [9], [12], [16], [58]. However, extrapolation of the earlier proposed baselines for some of the north European fisheries (e.g., [4], [11], [20]) might not be justified to at least some peripheral parts, where general human colonisation started relatively late – in the case of the current paper – only since the 15th century. As even the low level or ‘artisanal’ fishing can affect fish populations (e.g., [59]), and because some fish species like sturgeons and Atlantic salmon started already to decline in the Baltic Sea and adjacent river systems since the 12th century [9], [60], [61], the time-period discussed in the current work – late 17th century – cannot be taken as a true historic baseline for management of the whole Baltic Sea and adjacent freshwater systems, but rather as a signal on spatial heterogeneity in fisheries evolution timescales, driven by the timing of historical colonisation of areas of specific political interest or characterised by specific natural conditions.

### Conclusions

The current study provides quantitative evidence on fish catches in a coastal area of the north-eastern coast of the Baltic Sea at the end of the 17th century, for the period of the increased exploitation of fishery resources after the colonisation of the previously relatively sparsely populated areas. The archival sources enabled to estimate export amounts and assess the proportion of locally consumed fish in relation to the exports. Because the Baltic Sea is also inhabited by fish of freshwater origin, identifying the source of the fish–from freshwater or marine environment–posed challenges. These were solved by jointly using variety of information by including the nationality of fishermen, co-occurrence of different fish species in catches and fishing seasonality. It was concluded that most of the fish consumed and exported originated from the freshwater realm. The overall limited availability of archival material with relatively complete and reliable data for two years only did not allow us to perform any analysis on interannual variability and catch trends.

## Supporting Information

Table S1 **Information on the archival sources used in the current study.**
(DOC)
