# Supplementary material for: Quantification of the Early Small-Scale Fishery in the North-Eastern Baltic Sea in the Late 17th Century
Source: PLoS One. 2013 Jul 5;8(7):e68513. doi: 10.1371/journal.pone.0068513 (PMC3702608; doi:10.1371/journal.pone.0068513)
Supplement: Table S1 — Information on the archival sources used in the current study. (DOC) [file pone.0068513.s001.doc]

**Supporting information**

**Annex 1. Information on the archival sources used in the current study.**

| **Archival reference** | **Source** | **Content** | **Year** | **Folios** |
| --- | --- | --- | --- | --- |
| EAA 1646-2-342 | Journal, Der Einkommenen und Außgangen Schiffen unndt Schuten | Import Export | 1662 | 48 |
| EAA 1646-2-343 | Journal Der Einkommenen und Außgangen Schiffen, Schuten und Böhten | Import Export | 1666 | 48 |
| EAA 1646-2-344 | Journal Der Einkommenen und Außgangen Schiff[en] Schuten und Böhten | Import Export | 1668 | 47 |
| EAA 1646-2-345 | Journal Der Einkommenen und Außgangen Schiff[en] Schuten und Böhten | Import Export | 1671 | 41 |
| EAA 1646-2-346 | Journal Der Einkommenen und Außgangen Schiff[en] Schuten und Böhten | Import Export | 1672 | 38 |
| EAA 1646-2-347 | Journal Der Einkommenen Schiff[en] Schuten unnd Böhten; Journal Der Außgangenen Schiff[en] Schuten unnd Böhten | Import | 1677 | 38 |
| EAA 1646-2-348 | Journal Der Einkommenen Schiff[en] Schuten unnd Böhten; Journal Der Außgangenen Schiff[en] Schuten unnd Böhten | Import Export | 1679 | 53 |
| EAA 1646-1-1073 | [Summarised financial report for the year 1689, containing among others:] Narvens Uthgående Portorij Journael Pro Anno 1689; Narvens Inkommande Portorij Journal Pro Anno 1689 | Import Export different excises etc. | 1689 | unpaged |
| EAA 1646-2-350 | Lizent och Portorium J. Narven 1686–1690 | Different summarized financial reports, among others on confiscated fish products (1689) | 1689 | 146 |
| EAA 1646-1-1074 | [Summarised financial report for the year 1690, containing among others:] Narvens Inkommande Portorij Journall Pro Anno 1690; Narvens Uthgående Portorij Journal Pro Anno 1690 | Import Export Fish excise and other taxes | 1690 | unpaged |
| EAA 1646-1-1083 | Narfwens Uthgående Portory Journael Pro Anno 1694; Narvens Inkommande Portorij Journal Pro Anno 1694 | Import Export Fish excise and other taxes | 1694 | unpaged |
| EAA 1646-1-1086 | Narfwens Jnkommande Portorj Journael Pro Anno 1695; Narfwens Uthgående Portory Journael Pro Anno 1695 | Import Export Fish excise and other taxes | 1695 | unpaged |
| EAA 1646-2-351 | Liste über die von Narva ausgehenden Güter im Jahre 1696 | Summary of imported goods | 1696 | 1 |
| EAA 1646-2-352 | Stadtkassen Rechnungen betreffend den Hafen nebst Schiffsregister mit Angaben über die in Reval für Narva verzollten Waren. Portorium i Narven 1698 | Import from Reval (Tallinn) | 1698 | 14 |
| EAA 1646-2-349 | Narvens Inkommande Portory Journal Pro Anno 1699; Narfwens Uthgående Portory Journael Pro Ao. 1699 | Import Export and other taxes | 1699 | 104 |
| EAA 1646-2-353 | Schiffsregister (Narvasche Rechnung) betreffend die in Reval angekommenen und dort verzollten Waren. Bd. I. Anno 1702–1703 | Import from Reval (Tallinn) | 1702 | 11 |
| EAA 1646-2-354 | Schiffsregister (Narvasche Rechnung) betreffend die in Reval angekommenen und dort verzollten Waren. Bd. II. Anno 1703 | Import from Reval (Tallinn) | 1703 | 18 |
